# Therapy-Related B-cell Acute Lymphoblastic Leukemia: A Case Series and Literature Review

**DOI:** 10.7759/cureus.79664

**Published:** 2025-02-25

**Authors:** Hiba Asif, Usman Ahmad, Natalia Ahmad, Ammarah Tahir, Syed W Bokhari, Bushra Ahsan

**Affiliations:** 1 Hematology, Shaukat Khanum Memorial Cancer Hospital and Research Centre, Lahore, PAK; 2 Medical Oncology, Shaukat Khanum Memorial Cancer Hospital and Research Centre, Lahore, PAK; 3 Pathology and Laboratory Medicine, Shaukat Khanum Memorial Cancer Hospital and Research Centre, Lahore, PAK

**Keywords:** acute lymphoblastic leukemia (all), acute myeloid leukemia (aml), therapy-related leukemia, therapy-related myelodysplastic syndrome, therapy related myeloid neoplasms

## Abstract

Therapy-related B-cell acute lymphoblastic leukemia (B-ALL) is a rare disease associated with poor cytogenetics and inferior survival outcomes. In this case series, we present two cases of B-ALL following treatment for invasive ductal carcinoma of the breast and squamous cell carcinoma of the tongue, respectively, along with a third case of B-ALL arising in a patient with chronic lymphocytic leukemia after treatment. The most common cytogenetic abnormalities observed in these patients were t(9;22) and monosomy 7. This case series underscores the importance of recognizing therapy-related B-ALL as a distinct clinical entity, which plays a crucial role in ALL risk classification and the long-term management of both solid and hematologic cancer survivors.

## Introduction

Acute lymphoblastic leukemia (ALL) is a hematologic malignancy that arises from the malignant transformation, accumulation, and proliferation of early B- or T-lymphoid precursors in the bone marrow, blood, and lymphoid organs, leading to the suppression of normal hematopoiesis [1]. The majority of ALL cases occur as de novo malignancies [1]. However, a small subset arises due to predisposing factors such as genetic syndromes (e.g., Down syndrome, Fanconi anemia, and Bloom syndrome), exposure to ionizing radiation, chemotherapy, or infectious agents [2-5].

Therapy-related leukemias have become a significant complication of cytotoxic therapy (chemotherapy or radiotherapy) used in cancer treatment, posing a serious concern for both oncologists and cancer survivors. Among these, therapy-related myeloid neoplasms (t-MNs) are well recognized by WHO as a distinct entity, which includes therapy-related acute myeloid leukemia (t-AML) and therapy-related myelodysplastic syndrome. These conditions are associated with poor outcomes and inferior survival compared to de novo AML [6,7].

However, a small subset of patients develops therapy-related ALL (t-ALL) following cytotoxic therapy for a prior malignancy. Due to its rarity, t-ALL remains poorly understood, with only limited data available in the form of case reports and small case series. As a result, it has not yet been classified as a distinct entity like t-AML or other t-MNs. Patients with t-ALL often exhibit high-risk cytogenetic and molecular features and have poor prognoses [8].

Here, we describe three cases of B-cell acute lymphoblastic leukemia (B-ALL) that developed following treatment for primary malignancies, along with a literature review. To the best of our knowledge, no similar case series have been reported from our region.

## Case presentation

Case 1

A 42-year-old female presented to the clinic with a four-month history of a left breast lump. Her histopathology report confirmed Grade III invasive ductal carcinoma, triple-negative. A metastatic workup showed no evidence of disease spread. She underwent three cycles of 5-fluorouracil, epirubicin, and cyclophosphamide followed by three cycles of paclitaxel and radiation therapy. Her treatment was completed in 2015, and she remained on six-month follow-ups.

**Figure 1:**
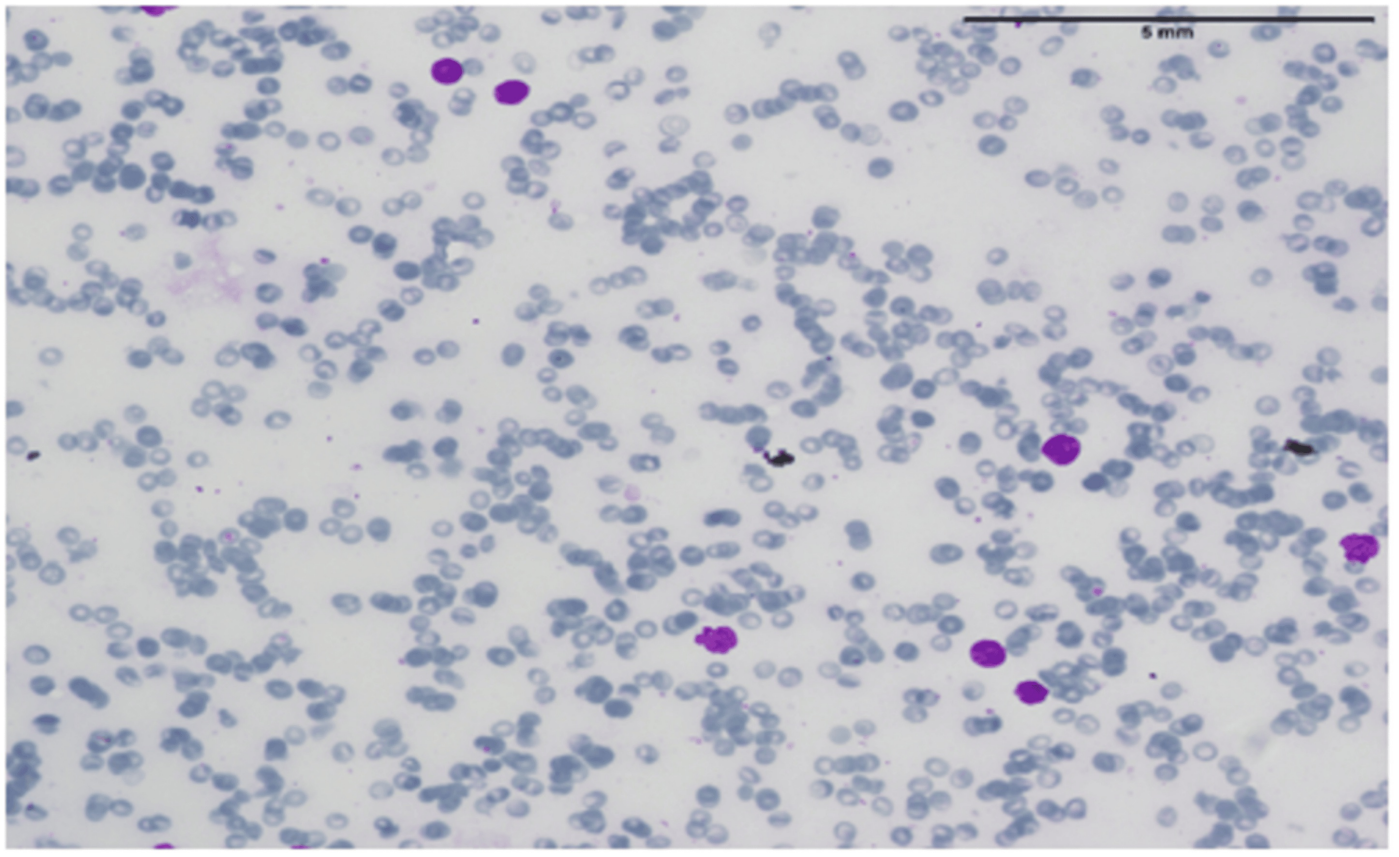
Bone marrow aspirate smear showing blast cells The diagnosis of acute leukemia is established when lymphoblasts constitute more than 20% of the bone marrow or peripheral blood smear.

**Figure 2:**
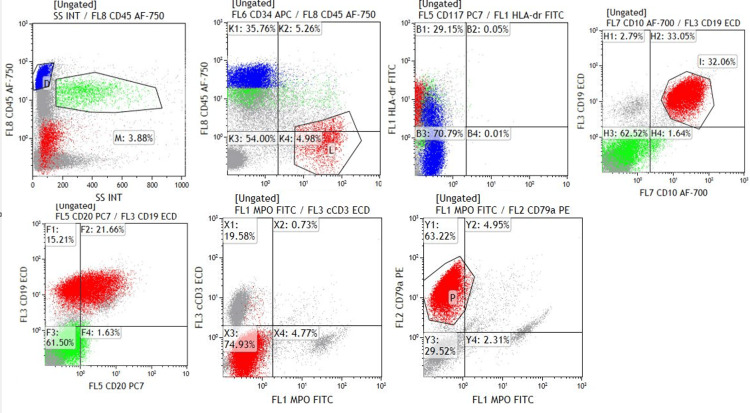
Immunophenotyping by flow cytometry showing blast cells (red population) expressing CD19, CD79a, CD34, CD10, and CD20

Three years after completing therapy, she presented to the ED with constipation and hemoptysis. Breast examination was unremarkable, and no other significant findings were noted on physical examination. CBC revealed hemoglobin (Hb) of 7 g/dL, total leukocyte count (TLC) of 48 × 10³/μL, platelets of 32 × 10³/μL, and 32% blast cells on morphology. A bone marrow biopsy showed diffuse infiltration with blast cells (Figure 1), and immunophenotyping by flow cytometry (Figure 2) confirmed the diagnosis of B-ALL. Fluorescence in situ hybridization (FISH) was positive for the BCR-ABL1 mutation, while cytogenetic analysis identified translocation t(9;22) and monosomy 7. She was diagnosed with t-ALL.

Chemotherapy for ALL was initiated promptly. She received Berlin-Frankfurt-Munich protocol-based induction chemotherapy, including daunorubicin, vincristine, PEG-asparaginase, cyclophosphamide, cytarabine, intrathecal methotrexate, and mercaptopurine. Unfortunately, she died approximately three months after induction due to treatment-related complications.

Case 2

A 36-year-old female with a diagnosed case of squamous cell carcinoma on the right side of the tongue underwent surgery (partial glossectomy) followed by radiation therapy to the face and neck (50 gray units in 20 fractions) in 2020. She remained on follow-up, with occasional visits to the emergency room for oral mucositis. Her disease remained in remission during this period.

Approximately one and a half years after completing treatment, in 2022, she presented to the ED with generalized weakness, myalgias, and a history of delayed wound healing following a tooth extraction. Physical examination revealed no lymphadenopathy or organomegaly. CBC showed Hb of 6.2 g/dL, TLC of 8 × 10³/μL, platelets of 56 × 10³/μL, and 52% blast cells on morphology. She was admitted for further evaluation of acute leukemia.

**Figure 3:**
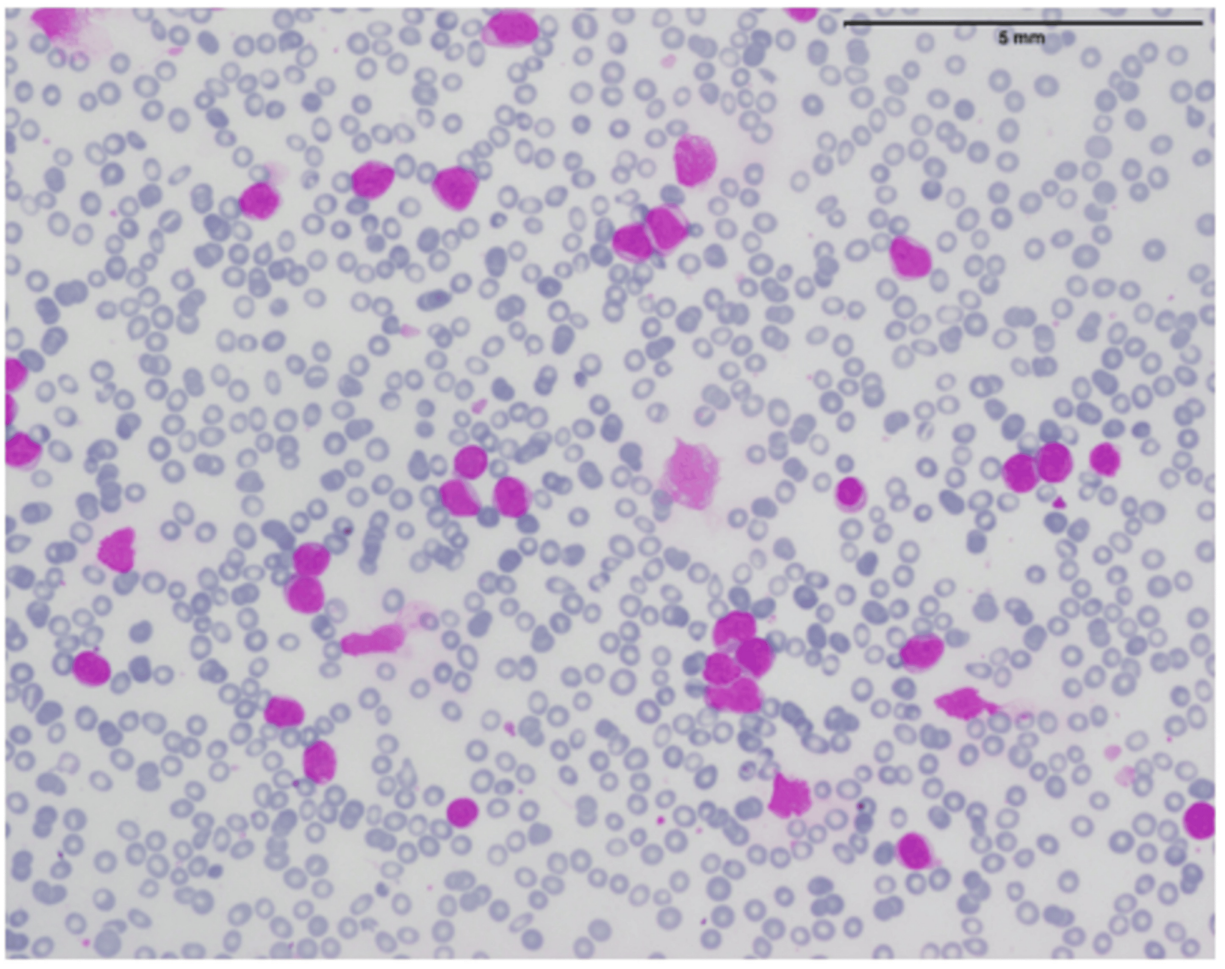
Bone marrow aspirate smear showing blast cells

**Figure 4:**
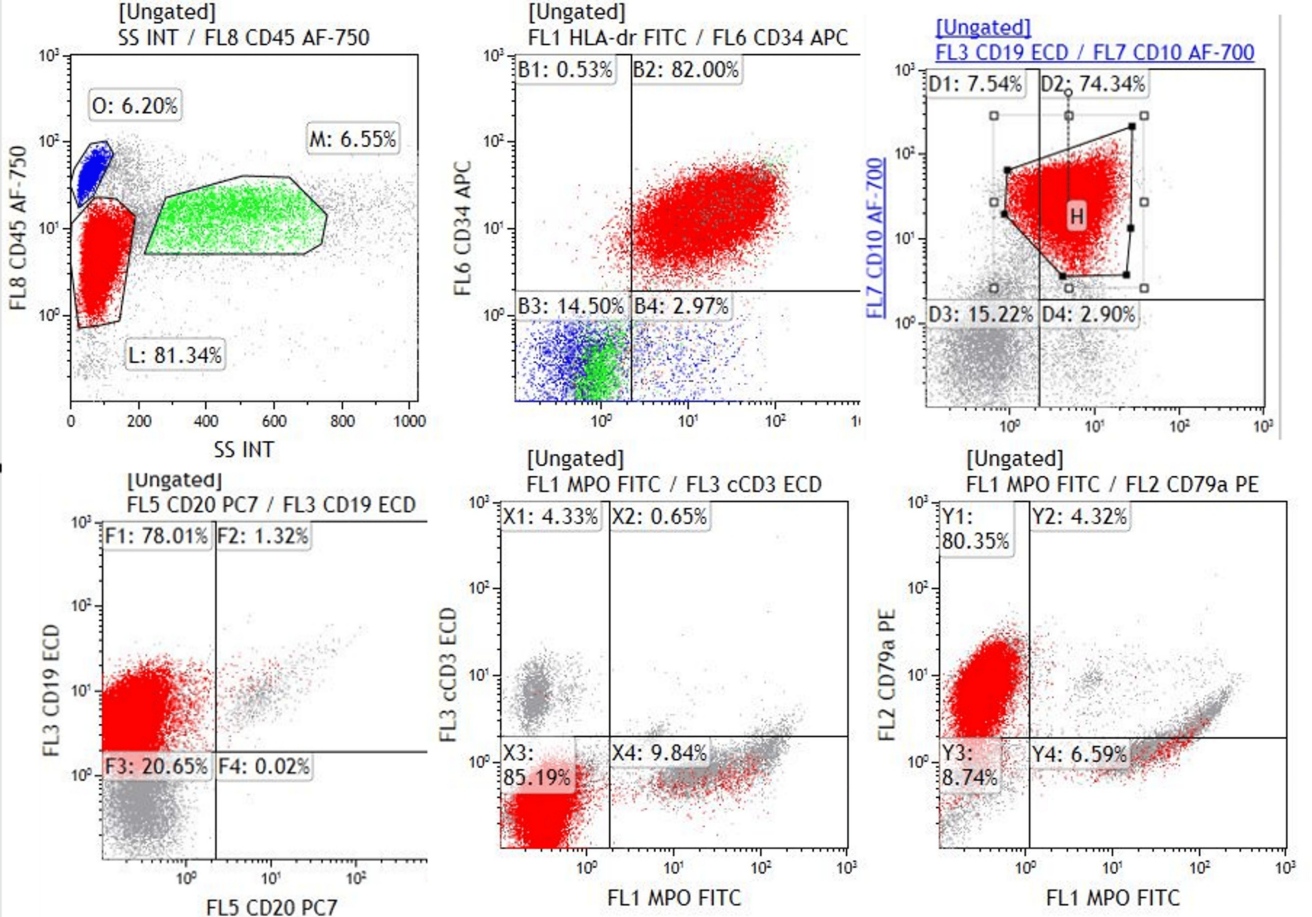
Immunophenotyping by flow cytometry The blast population, shown in red, expresses CD34, CD19, CD10, and CD79a while being negative for MPO and CD3.

Bone marrow aspirate revealed a predominant population of blast cells with diffuse infiltration on trephine biopsy (Figure 3). Immunophenotyping by flow cytometry confirmed B-ALL (Figure 4), while FISH was positive for the BCR-ABL1 fusion gene. The cytogenetic analysis identified t(9;22) and monosomy 7. She was diagnosed with B-ALL, possibly related to prior radiation therapy.

She was treated with the hyper-CVAD chemotherapy protocol (cyclophosphamide, vincristine, adriamycin, and dexamethasone) along with imatinib, a tyrosine kinase inhibitor. Following remission, she underwent a fully matched allogeneic bone marrow transplant. Unfortunately, she died seven months later due to transplant-related complications, specifically sepsis.

Case 3

A 50-year-old female presented with a four-month history of generalized weakness, neck swelling, and night sweats. Physical examination revealed splenomegaly and generalized lymphadenopathy. Peripheral blood counts showed lymphocytosis, and immunophenotyping by flow cytometry confirmed a diagnosis of chronic lymphocytic leukemia (CLL). As the patient was symptomatic, she received six cycles of fludarabine and cyclophosphamide, completing treatment in February 2019.

**Figure 5:**
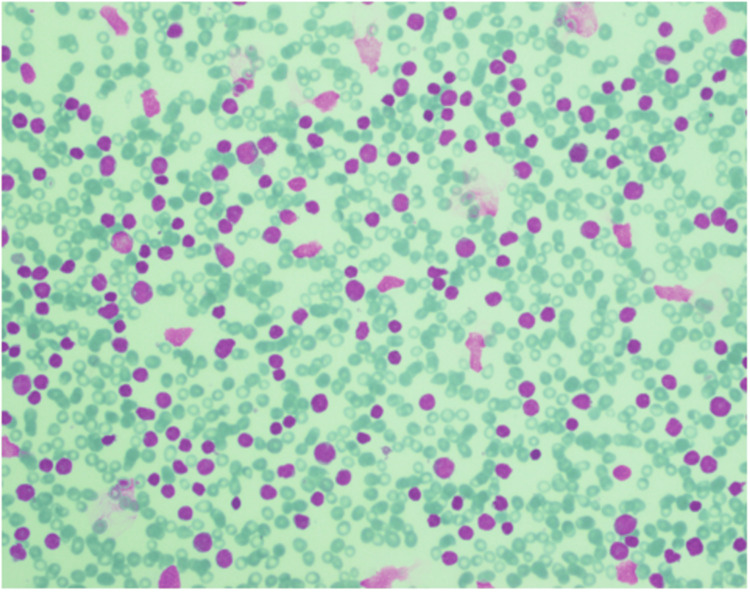
Bone marrow aspirate smear showing infiltration by small-sized blast cells

**Figure 6:**
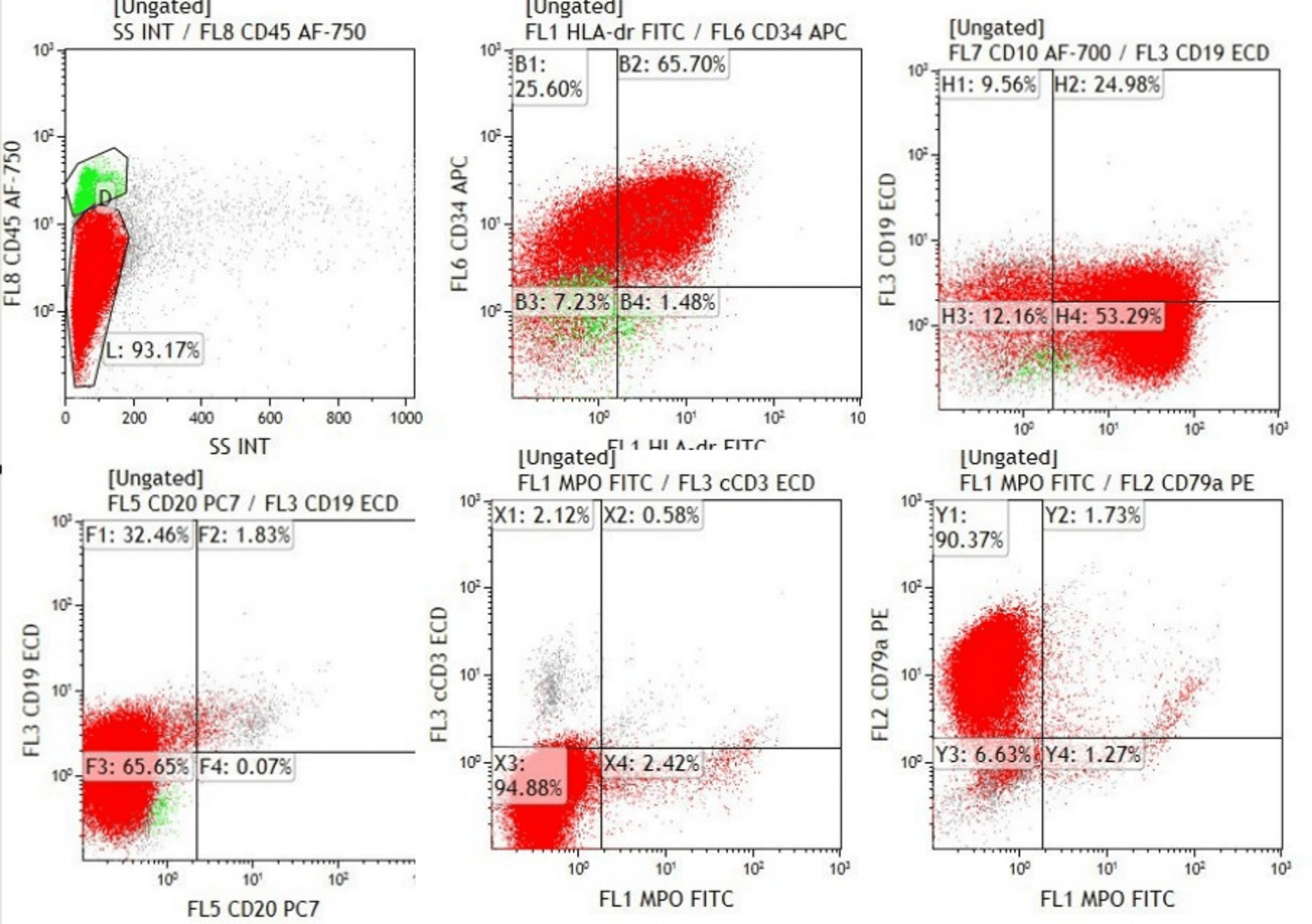
Flow cytometry plots showing blast cells expressing CD34, CD19, CD10, and CD79a, while being negative for cCD3 and MPO

One year after completing chemotherapy, she presented to the ED with fever, weight loss, and shortness of breath. CBC showed Hb of 8 g/dL, TLC of 5.5 × 10³/μL, platelets of 73 × 10³/μL, and 43% blast cells on morphology (Figure 5). Bone marrow examination revealed diffuse infiltration with blast cells, and immunophenotyping by flow cytometry confirmed B-ALL (Figure 6). Cytogenetic analysis identified translocations t(9;22) and t(7;16).

The patient’s condition deteriorated rapidly, and she was placed on supportive care. She died of septic complications within one month of diagnosis.

## Discussion

t-ALL is defined as ALL that develops in patients who have previously received cytotoxic treatments, such as chemotherapy or radiotherapy, for solid or hematological malignancies [9]. Unlike t-AML and t-MNs, t-ALL is not currently recognized as a distinct entity by WHO classification. It is an uncommon disease and is considered an independent risk factor for poor prognosis, with outcomes worse than those of de novo ALL [8].

t-ALL accounts for approximately 3-9% of adult ALL cases [8] and is significantly less common than t-MNs. While the exact pathophysiology of t-ALL is not as well understood as that of t-MNs, it is believed that certain cytotoxic therapies - such as alkylating agents, topoisomerase II inhibitors, and ionizing radiation - exert genotoxic effects on hematopoietic cells, leading to DNA damage and chromosomal abnormalities [9,10].

In our study, we identified three cases of t-ALL. The first two cases involved patients with a history of invasive ductal carcinoma of the breast and squamous cell carcinoma of the tongue, respectively, who developed B-ALL following treatment. The breast cancer patient had received both chemotherapy and radiotherapy, while the tongue cancer patient had undergone radiotherapy alone, making both cases consistent with t-ALL. Among solid tumors, breast carcinoma is the most common antecedent malignancy due to increased exposure to both chemotherapy (alkylating agents and topoisomerase II inhibitors) and radiotherapy. Among hematologic malignancies, lymphomas and other lymphoproliferative disorders are the most frequent precursors to t-ALL [9].

Common cytogenetic abnormalities in t-ALL include 11q23 (KMT2A) rearrangement, the Philadelphia chromosome, monosomy 5, 7, or 17, hypodiploidy, and complex karyotypes [9-12]. The 11q23 rearrangement is particularly associated with prior exposure to topoisomerase II inhibitors and is linked to a shorter latency period before leukemia onset [9,12].

Our third case is a rare presentation of B-ALL occurring in a patient with CLL after one year of purine analog-based chemoimmunotherapy. CLL is a mature B-cell disorder characterized by the accumulation of functionally incompetent clonal B lymphocytes. Disease transformation and secondary malignancies are serious complications of CLL, with approximately 5% of treated CLL patients developing therapy-related myeloid neoplasms (AML/MDS) as secondary hematologic malignancies [13].

In contrast, B-ALL occurring in CLL patients is extremely rare and remains poorly characterized. Few cases have been reported, and the underlying pathophysiology remains unclear. There is conflicting data on whether ALL arises from the same B-cell clone as CLL or represents a separate secondary malignancy induced by therapy. In a literature review by Zarrabi et al. in 1977 [14], 31 cases of acute leukemia were reported in CLL patients, including 10 cases of lymphoblastic leukemia; however, none of these cases were assessed for clonal relationships between ALL and CLL. Chakhachiro et al. [15] reported four cases and concluded that B-ALL in CLL patients likely represents a clonally unrelated secondary neoplasm. Conversely, some studies have described clonal evolution from CLL to B-ALL, supported by immunoglobulin heavy-chain variable region (IGHV) gene mutation analysis [13,16].

IGHV mutation analysis was not performed for our patient due to the unavailability of the test at our center, so we categorized this case as secondary ALL, most likely therapy-related. Notably, all three cases in our study developed ALL after treatment, with B-ALL being the common phenotype, as summarized in Table 1.

**Table 1:** Disease-related characteristics of t-ALL patients ALL, acute lymphoblastic leukemia; B-ALL, B-cell acute lymphoblastic leukemia; BMA, bone marrow aspirate; CLL, chronic lymphocytic leukemia; Hb, hemoglobin; t-ALL, therapy-related acute lymphoblastic leukemia

Variables	Case 1	Case 2	Case 3
Age at presentation (year)	42	36	50
Gender	Female	Female	Female
Marital status	Married	Married	Married
Primary malignancy	Invasive ductal carcinoma of the breast	Squamous cell carcinoma of the tongue	CLL
Previous history of chemotherapy/radiotherapy exposure	Yes	Yes	Yes
Transformed to	ALL	ALL	ALL
Latency period	3 years	1.5 years	1 year
Hb (g/dl)	7	6.2	8
Platelets (×10³/µL)	32	56	73
Peripheral blood blast percentage (%)	32	52	43
Blast percentage on BMA	38	90	70
Immunophenotyping by flow cytometry	B-ALL	B-ALL	B-ALL
Molecular studies	BCR-ABL	BCR-ABL	-
Cytogenetics	t(9;22), monosomy 7	t(9;22), monosomy 7	t(9;22), t(7;16)
Disease outcome	Died after three months of diagnosis	Died after seven months of diagnosis	Died after one month of diagnosis

All three patients had t(9;22) on karyotyping, with two also exhibiting monosomy 7, indicating that these are common cytogenetic abnormalities in t-ALL. Given their association with poor prognosis, these abnormalities may have contributed to the unfavorable outcomes observed. All three patients died within a few months of diagnosis - two due to disease-related complications and one due to transplant-related complications. Overall survival in t-ALL is significantly worse than in de novo ALL; however, allogeneic bone marrow transplantation in eligible patients may improve outcomes [8].

A key limitation of our study is that it is a single-center analysis involving a small patient population, which may not be representative of national data. Larger, more extensive studies are needed to further characterize t-ALL as a distinct clinical entity.

## Conclusions

t-ALL is an uncommon disease but is associated with a poor prognosis. Given its continued occurrence, we suggest that large cohort studies be conducted to establish t-ALL as a distinct entity in WHO classification. Additionally, further exploration through molecular studies may provide deeper insights into the disease and its clinical implications, ultimately aiding in better risk stratification and management.
